# Evolutionary patterns of carbohydrate transport and metabolism in *Halomonas boliviensis *as derived from its genome sequence: influences on polyester production

**DOI:** 10.1186/2046-9063-8-9

**Published:** 2012-04-17

**Authors:** Daniel Guzmán, Andrea Balderrama-Subieta, Carla Cardona-Ortuño, Mónica Guevara-Martínez, Nataly Callisaya-Quispe, Jorge Quillaguamán

**Affiliations:** 1 Centro de Biotecnología, Facultad de Ciencias y Tecnología, Universidad Mayor de San Simón, Cochabamba, Bolivia; 2 Department of Biotechnology, Lund University, P.O. Box 124, SE-221 00 Lund, Sweden

**Keywords:** *Halomonas boliviensis*, Halophilic bacterium, *Halomonas*, *Halomonadaceae*, Biopolyesters, Polyhydroxyalkanoates, Genome evolution, Population genetics

## Abstract

**Background:**

*Halomonas boliviensis *is a halophilic bacterium that is included in the γ-Proteobacteria sub-group, and is able to assimilate different types of carbohydrates. *H. boliviensis *is also able to produce poly(3-hydroxybutyrate) (PHB) in high yields using glucose as the carbon precursor. Accumulation of PHB by microorganisms is induced by excess of intracellular NADH.

The genome sequences and organization in microorganisms should be the result of evolution and adaptation influenced by mutation, gene duplication, horizontal gen transfer (HGT) and recombination. Furthermore, the nearly neutral theory of evolution sustains that genetic modification of DNA could be neutral or selected, albeit most mutations should be at the border between neutrality and selection, i.e. slightly deleterious base substitutions in DNA are followed by a slightly advantageous substitutions.

**Results:**

This article reports the genome sequence of *H. boliviensis*. The chromosome size of *H. boliviensis *was 4 119 979 bp, and contained 3 863 genes. A total of 160 genes of *H. boliviensis *were related to carbohydrate transport and metabolism, and were organized as: 70 genes for metabolism of carbohydrates; 47 genes for ABC transport systems and 43 genes for TRAP-type C4-dicarboxylate transport systems. Protein sequences of *H. boliviensis *related to carbohydrate transport and metabolism were selected from clusters of orthologous proteins (COGs). Similar proteins derived from the genome sequences of other 41 archaea and 59 bacteria were used as reference. We found that most of the 160 genes in *H. boliviensis*, c.a. 44%, were obtained from other bacteria by horizontal gene transfer, while 13% of the genes were acquired from haloarchaea and thermophilic archaea, only 34% of the genes evolved among Proteobacteria and the remaining genes encoded proteins that did not cluster with any of the proteins obtained from the reference strains. Furthermore, the diversity of the enzymes derived from these genes led to polymorphism in glycolysis and gluconeogenesis. We found further that an optimum ratio of glucose and sucrose in the culture medium of *H. boliviensis *favored cell growth and PHB production.

**Conclusions:**

Results obtained in this article depict that most genetic modifications and enzyme polymorphism in the genome of *H. boliviensis *were mainly influenced by HGT rather than nearly neutral mutations. Molecular adaptation and evolution experienced by *H. boliviensis *were also a response to environmental conditions such as the type and amount of carbohydrates in its ecological niche. Consequently, the genome evolution of *H. boliviensis *showed to be strongly influenced by the type of microorganisms, genetic interaction among microbial species and its environment. Such trend should also be experienced by other prokaryotes. A system for PHB production by *H. boliviensis *that takes into account the evolutionary adaptation of this bacterium to the assimilation of combinations of carbohydrates suggests the feasibility of a bioprocess economically viable and environmentally friendly.

## Background

Cellular evolution and adaptation have imprinted patterns in microbial genomes through mutation, gene duplication, horizontal gen transfer (HGT) and recombination [1,2]. The genomes of microorganisms of the three domains of life have experienced such genetic modifications to succeed on their permanence in a particular habitat, where environmental conditions and the size of the microbial populations might influence the organization and number of genes in a particular species throughout the time [1,3]. Furthermore, the nearly neutral theory of evolution points out that genetic modification of DNA could be neutral or selected, albeit most mutations should be at the border between neutrality and selection, i.e. slightly deleterious base substitutions in DNA are followed by a slightly advantageous substitutions [1].

The increasing number of genome sequences of different organisms is helping to discern how microbial species diverged. Recent reports on the evolutionary traits followed by different bacteria and archaea have demonstrated that the transfer of genes among these organisms, also referred as horizontal gene transfer, has led to net-like relationships among their genomes [2,4,5]. Nevertheless, the phylogenetic association among prokaryotes derived from the sequences of proteins encoded by 102 different genes was consistent to the taxonomic differentiation observed when 16 rRNA sequences of microorganisms are analyzed [4]. The 102 proteins were mainly related to translation and transcription, although proteins involved in the transport and metabolism of amino acids, metal ions and carbohydrates revealed such taxonomic information as well [4].

The aforementioned studies included the genome sequences of extremely halophilic archaea such as *Haloarcula marismortui*, *Haloquadratum walsbyi *and a *Halobacterium *sp. [4]. These studies on the genome sequences did not include halophilic bacteria. However, a report on the genes of poly(3-hydroxybutyrate) (PHB) polymerases, PHB depolymerases and ectoine synthesis by *Halomonas *sp. TD01, a halophilic bacterium, suggested that HGT has a role to play on the genome organization of the microorganism [6]. Halophilic microorganisms require salt (NaCl) to grow; a halophile should grow optimally at NaCl concentrations of 5% (w/v) or higher, and tolerate at least 10% (w/v) salt [7]. There are five genome sequences of halophilic bacteria available in public data bases. The sequences of *Chromohalobacter salexigens *and *Halorhodospira halophila *were first published followed by the sequence of *Halomonas elongata *[8,9], *Halomonas *sp. TD01 [6] and *Halomonas *sp. HAL1 [10]. *Chromohalobacter *and *Halomonas *species are included in the family *Halomonadaceae *within the γ-Proteobacteria subgroup. The family *Halomonadaceae *contains only halophilic and halotolerant aerobic heterotrophs; some of them are able to grow in media with up to 30% (w/v) NaCl [7]. Halophilic bacteria maintain low concentrations of salt intracellularly by accumulating organic compounds of low molecular weight, also known as osmolytes or "compatible solutes" such as ectoine [11].

Understanding the evolution and levels of polymorphism among genes is attracting much attention in evolutionary biology and biotechnology. Evolution of energy-producing pathways, particularly glycolysis and gluconeogenesis, posses relevance since they determine the type of carbon sources that a species is able to assimilate, and link to metabolic routs that may generate compounds of biotechnological interest [12]. Theories on the evolution of the metabolisms of organisms consider that enzyme polymorphism--alleles for the different enzymes or allozymes--in metabolic pathways was related to genetic mutations [12-14]. A proposal states that the fitness of the pathways associated with an increasing flux is influenced by selected mutations of genes that enhance enzyme activities, albeit enzyme improvements do not continue indefinitely [12,14]. Mutations will reach a point at which the incremental gains of fitness for a new mutation will be equaled by the noise caused by the random genetic variation [12,14]. At this stage, the genes or enzymes might evolve under a nearly neutral trend [12,14]. Moreover, metabolic control in the organisms is also to regulate molecular evolution as well [12,14]. The proposal assumes no contextual changes such as a change in the functional conditions of an enzyme originated by either epistasis or the environment; or a change in the effective population size of the species [12].

*Halomonas boliviensis *is a halophilic bacterium that can develop under a wide range of NaCl concentrations (i.e. 0-25% (w/v)), pH (5-11) and temperatures (0-45°C) [15]. It can also assimilate several carbohydrates as carbon source for growth [15]. Bioprocesses have been designed to attain high productivities of a polyester and osmolytes by *H. boliviensis *using glucose as the carbon precursor [16,17]. The polyester accumulated by the bacterium is poly(3-hydroxybutyrate) (PHB), which is used as carbon and energy reservoir [18]. PHB is synthesized by several bacteria from acetyl-CoA when an excess of NADH is present in the bacterial cytoplasm [19]. Such excess can be generated when a high concentration of a carbon source is added to a culture medium and cell growth is limited by the depletion of an essential nutrient, e.g. nitrogen, oxygen, trace elements among others [19]. PHB is attracting much attention in biotechnology because it is a biodegradable plastic-like material, and possesses potential in biomedical applications such as tissue engineering, organ transplants and drug delivery systems [20]. Moreover, the efficiency and economics of the manufacturing process of PHB are determined by the carbon source, fermentation process, and downstream processing of the polymer. The development of cultivation conditions for microorganisms that allow high PHB content and productivity from cheap and renewable carbon sources is therefore important [21,22].

The present research work reports the genome sequence of *H. boliviensis*. It also depicts the evolutionary trends that proteins of *H. boliviensis *have experienced to allow the transport of carbohydrates and their assimilation to achieve acetyl-CoA. The conclusions drawn from these studies were used to create an alternative production system of PHB by *H. boliviensis *using a combination of carbohydrates. This system should lead to a more economically and environmentally beneficial bioprocess.

## Methods

### Genome sequencing

The fine high coverage genome sequence, gene prediction, repetitive sequence, COGs and KEGG annotation of *Halomonas boliviensis *LC1^T ^(= DSM 15516^T^) were obtained at BGI-Hongkong Co., Hong Kong. For this, Illumina HiSeq 2000 technology was used to conduct paired-end sequencing for DNA samples, and constructed a 1,000 bp library with extended data of 500 Mb. Genome coverage based on k-mer was 95.4%, and genome coverage based on reads mapping was 99.9%. Glimmer 3.0 software package was used to conduct *de novo *gene prediction [23]. The functional annotation was accomplished by analysis of protein sequences. Genes of *H. boliviensis *were aligned to others in databases to attain its corresponding functional annotation. To ensure the biological meaning, only one high-quality information as annotation to the genes from many results was chosen. BLAST was used to accomplish functional annotation combined with different databases. BLAST version: blastall 2.2.21 software (provided by the National Center for Biotechnology Information, NCBI) was used for these studies. Alignment results were obtained using the following databases: KEGG, COG, SwissProt, TrEMBL, NR. This whole genome shotgun project was deposited at DDBJ, EMBL and GenBank under the accession number AGQZ00000000. The version described in this paper is the first version, AGQZ01000000.

### Evolutionary analysis

A total of 6,901 alignments of clusters of orthologous proteins (COGs) of 59 bacteria and 41 archaea, as classified in COGs [24] and EggNOG [25] data bases, were gently provided by Puigbò, Wolf and Koonin (2009). The protein sequences of these 100 microorganisms were used as reference for the evolutionary analysis. Protein sequences of *H. boliviensis *related to carbohydrate transport and metabolism were selected and aligned along with the references for each corresponding COG (Additional file 1: Table S1, supplementary data) using the Muscle program [26] included in the MEGA 5 software package [27] with default parameters. Unrooted maximum likelihood phylogenetic trees were constructed using MEGA 5 under a WAG with frequencies (+F) model, with uniform mutation rates among amino acid sites and complete deletion of gaps and missing data.

### Analysis and assembly of supernetworks

Supernetworks were constructed by combining the phylogenetic trees of proteins of the glycolysis and gluconeogenesis metabolisms in *H. boliviensis *and reference strains using the SplitsTree4 program [28,29] with default parameters. Three analyses were performed for these studies: 1) A supernetwork obtained from three COGs (0126, 0149 and 0837). Both COG0126 and COG0149 are considered among the 102 genes that contain taxonomic information that discriminate well bacteria and archaea in already known families and genera [4]; 2) A supernetwork obtained after combining six COGs (0126, 0149, 0837, 0469, 0696 and 837); and 3) A supernetwork obtained after combining twenty two COGs (0057, 0126, 0148, 0149, 0166, 0191, 0205, 0235, 0365, 0469, 0508, 0696, 0837, 1012, 1063, 1109, 1249, 1454, 1866, 2017, 2609 and 4993). Supernetworks were analyzed according to method described by Huson *et al. *in 2006 [28].

### Culture media composition

Seed culture and PHB production media were formulated as described previously [16]. Seed culture contained% (w/v): NaCl, 2.5; MgSO_4_•7H_2_O, 0.25; K_2_HPO_4_, 0.05; NH_4_Cl, 0.23; FeSO_4_•7H_2_O, 0.005; sucrose 1; monosodium glutamate (MSG), 0.3 and TRIS, 1.5. The PHB production medium included % (w/v): NaCl, 2.5; MgSO_4_•7H_2_O, 0.5; K_2_HPO_4_, 0.22; NH_4_Cl, 0.4; FeSO_4_•7H_2_O, 0.005; MSG, 0.2; and the following concentration of carbohydrates % (w/v): 1) 2.5 sucrose, 2) 2.0 sucrose and 0.5 glucose, 3) 1.5 sucrose and 1 glucose, 4) 1.0 sucrose and 1.5 glucose, 5) 0.3 sucrose, 0.7 glucose and 1.5 dried molasses and 6) 2.5 dried molasses for 6 different assays, respectively. The composition of the molasses used was 78.1% sucrose, 15.3% glucose and 6.6% of other uncharacterized solids. A low amount of MSG is added to the production medium to induce its depletion by *H. boliviensis *during the cultivation.

### *H. boliviensis *growth and PHB production in flasks

*H. boliviensis *was grown in 100 ml of seed culture medium in 1,000-ml flasks with rotary shaking at 220 rpm, 30°C for 13 h. The pH of the medium was adjusted to 7.5 using concentrated HCl. Subsequently, 5 ml of the seed culture were inoculated in 1,000-ml Erlenmeyer flasks containing 95 ml of PHB production medium. The pH of the PHB production medium was initially adjusted to 7.5 using 5 M NaOH. The cultures were incubated at 35°C with shaking at 220 rpm, and samples were withdrawn at different time intervals during the cultivation.

### Quantitative analyses

Cell dry weight (CDW) and PHB content in *H. boliviensis *were determined as reported previously [18]. Residual cell mass (RCM) concentration was calculated as the difference between the CDW and PHB concentration, while PHB content (wt%) was obtained as the percentage of the ratio of PHB concentration to the CDW as defined by Lee *et al. *in 2000 [30]. All analyses were performed in triplicate.

Glutamate concentration was determined by high performance liquid chromatography (HPLC) analysis, as described previously [31], using a Perkin-Elmer HPLC system with an Aminex HPX-87 C column (Biorad) and a UV detector at 65°C. Calcium chloride solution (5 mM) was used as mobile phase at a flow rate of 0.5 ml/min. Glutamate was monitored at 210 nm. Glucose and sucrose were determined using the same HPLC system with a Polypore CA column (Perkin-Elmer), a RI detector at 80°C and water as mobile phase at a flow rate of 0.3 ml/min.

## Results and discussion

### Genome of *H. boliviensis*

Table 1 provides a description of the genome composition of *H. boliviensis*. The chromosome size of *H. boliviensis *(4 119 979 bp) was slightly longer than those determined for *H. elongata *(4 061 296 bp) [9], *Halomonas *sp. TD01 (4 092 837 bp) [6] and *Chromohalobacter salexigens *(3 696 649 bp) (Accession number: CP000285.1). The % of G+C content showed in Table 1 is similar to that determined experimentally for *H. boliviensis*, i.e. 52.6% [15], and is lower than that found for the genome of *H. elongata *(63.6%) and that evaluated for the description of *C. salexigens *(64.2%) [32]. Such wide difference between the G+C content of different *Halomonas *and *Chromohalobacter *species is a feature of the family *Halomonadaceae *[33]. Moreover, the genes constitute most part of the chromosome of *H. boliviensis *and the %G+C content for the region containing the genes was similar to that found in its chromosome (Table 1). On the other hand, the number of genes in the genome of *H. boliviensis *(3 863) is slightly higher than that reported for *H. elongata *(3 555) [9].

**Table 1:** Genome of *H.boliviensis*

Chromosome	1
DNA, total number of bases % G+C content	4 119 979 54.69
Number of genes	3 863
Length occupied by genes (bp)	3 673 824
% G+C content in the gene region	55.64
% Gene/Chromosome	89.17
Length occupied by the intergenic region (bp)	446 146
% G+C content in the intergenic region	46.86
% Intergenic length/Chromosome	10.82

### Inferring the evolution of proteins involved in the uptake and metabolism of carbohydrates

Protein sequences of *H. boliviensis *related to carbohydrate transport and metabolism were obtained from clusters of orthologous proteins (COGs), as classified in COGs and EggNOG data bases [24,25]. A total of 160 genes of *H. boliviensis *encoded proteins for these clusters: 70 genes were related to the metabolism of carbohydrates; 47 genes were related to ABC transport systems and encoded 14 permease proteins, 23 ATPase proteins and 10 periplasmic proteins; and 43 genes were related to TRAP-type C4-dicarboxylate transport systems and encoded 15 large permease proteins, 11 small permease proteins and 17 periplasmic proteins (Additional file 1: Table S1, supplementary data). Similar proteins were selected from COGs derived from the genome sequences of other 41 archaea and 59 bacteria. To perform evolutionary analyses, unrooted phylogenetic trees were constructed based on a maximum likelihood approach using the sequences of the proteins of *H. boliviensis *and proteins of other 100 microorganisms for each corresponding COG.

**Figure 1:**
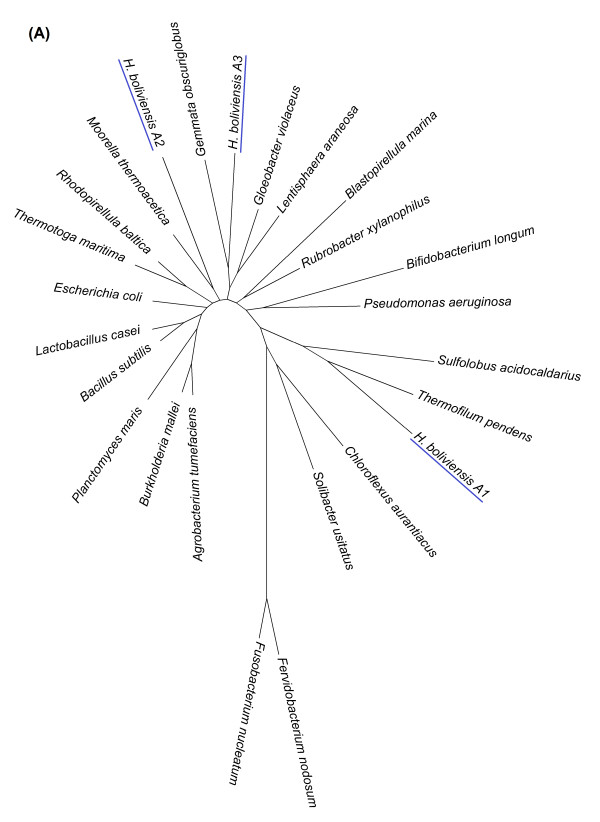
**Phylogenetic tree relating clusters of orthologous protein sequences (COGs):ABC type transport system for ribose, xylose, arabinose and galactoside, COG1172**. The tree was constructed under a maximum likelihood approach using MEGA 5 software and the WAG with frequencies (+F) model. GeneBank accession numbers of the sequences included in the figure are provided in Additional file 2: Table S2.

Figure 1 presents three phylogenetic trees that were selected to exemplify the genetic modifications experienced by the genome of *H. boliviensis*. Figure 1 shows a phylogenetic tree for a COG related to the ABC type transport system for ribose, xylose, arabinose and galactoside. *H. boliviensis *has three alternative forms of genes, i.e. alleles, for this tree. The first allele (*H. boliviensis *A1) was clustered with thermophilic archaea (Figure 1), hence implying a long distance HGT [5]. After comparing the closest identities of this allele to other sequences in pubic data bases, we found that the sequence corresponded to a periplasmic binding protein. We also found that the amount of acidic amino acids of *H. boliviensis *A1 (10.1% of the total residues in the protein) was significantly higher than the basic amino acids (3.5%), resulting in a ratio of 2.9 of acidic to basic amino acids. Various scientific articles reported that some extracellular and periplasmic enzymes of halophilic bacteria tolerate high temperatures, and possess a relatively high content of acidic amino acids [8,34]. The result obtained in Figure 1 denotes that *H. boliviensis *had originally obtained the binding protein from a thermophile and it diverged later. The second allele (*H. boliviensis *A2) was also identified as a periplasmic binding protein with similar functions to those of the first allele. However, the second allele shared a closer affiliation to proteins of bacteria other than Proteobacteria (Figure 1). The ratio of acidic (9.4% of the total residues in the protein) to basic (5.5%) amino acids for allele 2 was 1.7. *H. boliviensis *is a microorganism that is able to grow at low (0°C) and high (45°C) temperatures, whereby it should be useful for this organism to hold two different proteins that can accomplish a similar task; one active at low temperatures and the second active at high temperatures. The third allele (*H. boliviensis *A3) was clustered along with trans-membrane proteins of Planctomycetes, Cyanobateria and Lentisphaerae (Figure 1). Both allele 2 and 3 suggest HGT among bacteria [5].

**Figure 2:**
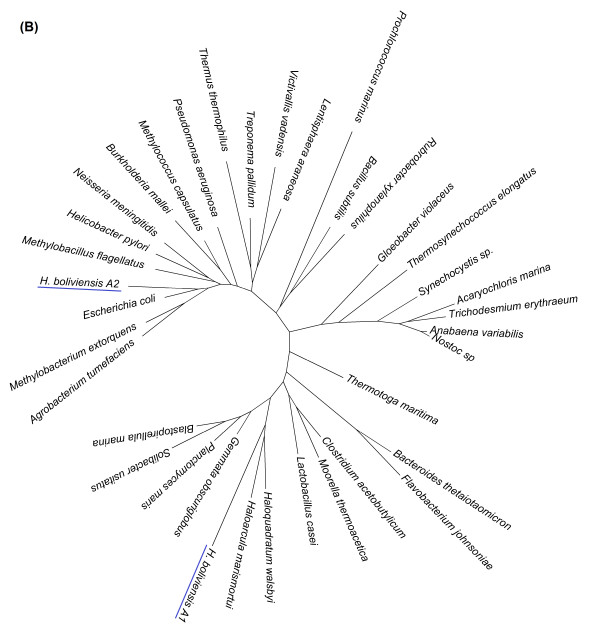
**Phylogenetic tree relating clusters of orthologous protein sequences (COGs): keto-3-deoxy-6-phosphogluconate aldolases, COG0800**. The tree was constructed under a maximum likelihood approach using MEGA 5 software and the WAG with frequencies (+F) model. GeneBank accession numbers of the sequences included in the figure are provided in Additional file 2: Table S2.

**Figure 3:**
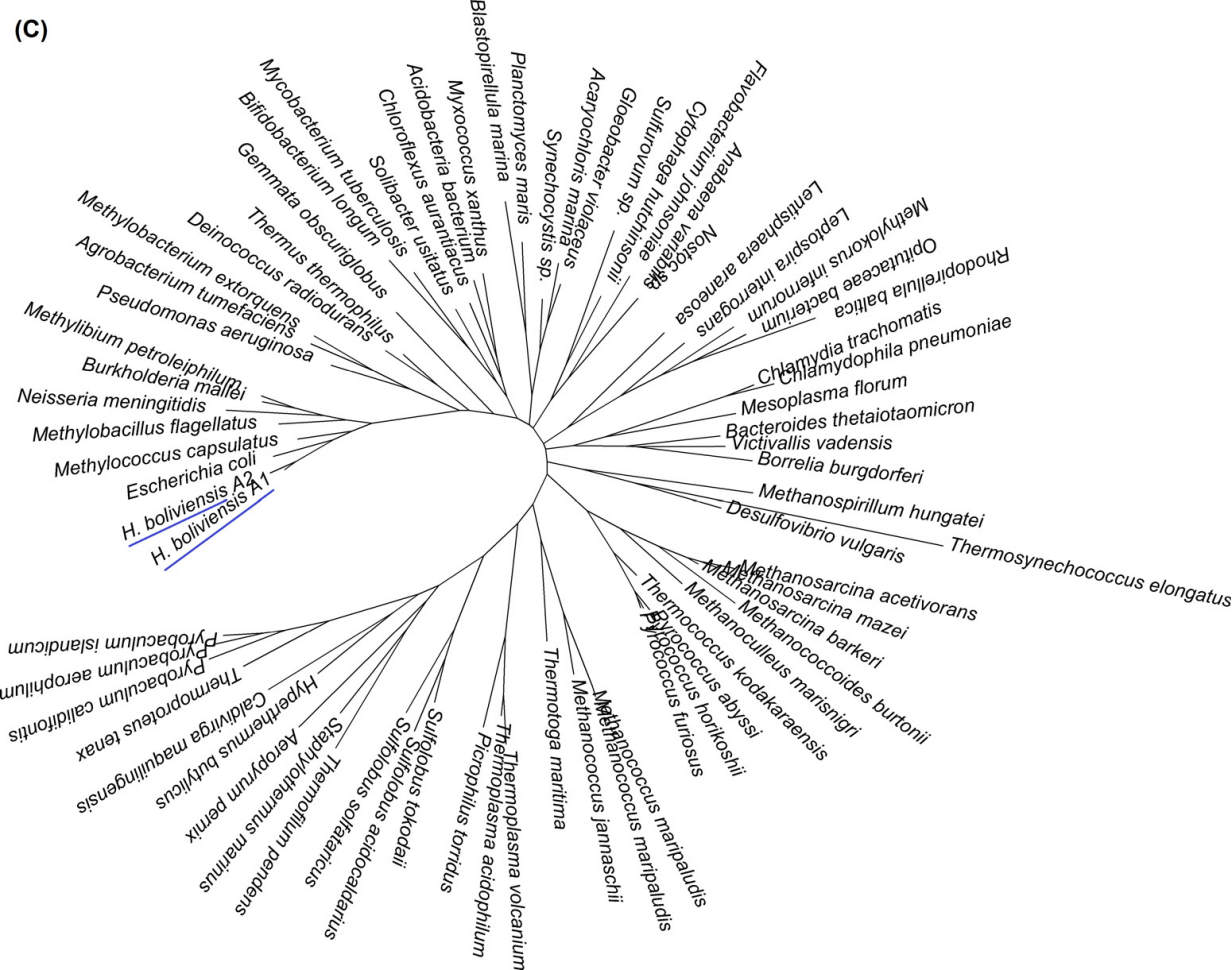
**Phylogenetic tree relating clusters of orthologous protein sequences (COGs): Pyruvate kinases, COG0469**. The tree was constructed under a maximum likelihood approach using MEGA 5 software and the WAG with frequencies (+F) model. GeneBank accession numbers of the sequences included in the figure are provided in Additional file 2: Table S2.

**Figure 4:**
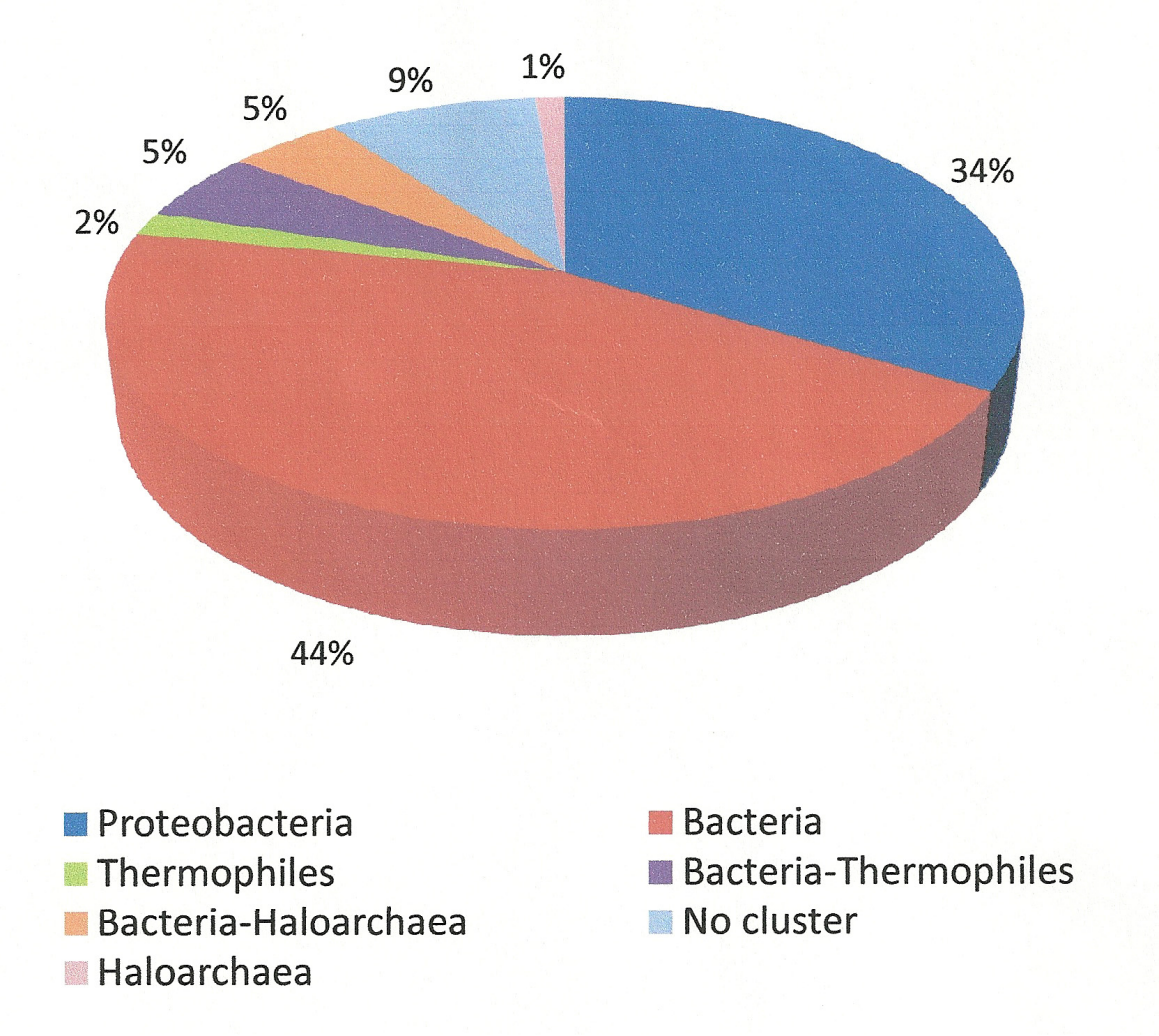
**Percentage of proteins that *H. boliviensis *obtained from other bacteria, halophilic archaea and thermophilic archaea by horizontal gene transfer (HGT), and proteins that evolved among proteins of other Proteobacteria**. Some proteins of *H. boliviensis *did not form a cluster with any of the proteins of the microorganisms used as reference. The proteins analyzed were related to COGs of carbon transport and metabolism as listed in Additional file 1: Table S1.

On the other hand, Figure 2 depicts a phylogenetic tree for a COG corresponding to keto-3-deoxy-6-phosphogluconate aldolases. The first allele of *H. boliviensis *in this phylogenetic tree was closely related to proteins of halophilic archaea, while the second diverged among enzymes of Proteobacteria with a close relationship to γ-Proteobacteria (Figure 2). *H. boliviensis *has also adapted to a wide range of NaCl concentrations. At different NaCl concentrations, *H. boliviensis *should thrive in environments with distinct type of microorganisms varying from non-halophilic to extreme halophilic archaea [7]. *H. boliviensis *showed to be able to acquire genes from other organisms that share its habitat (Figures 1, 2). Moreover, Figure 3 depicts two alleles that are closely related to proteins of γ-Proteobacteria, as might be expected. The same evolutionary analyses were followed with all COGs related to carbohydrate transport and metabolism. The results obtained are summarized in Additional file 1: Table S1 and in Figure 4. Interestingly, most of the proteins (44%) of *H. boliviensis *involved in carbohydrate transport and metabolism were obtained from other bacteria; only a 34% of the proteins evolved among Proteobacteria (Figure 4). Moreover, transfer of genes from thermophilic organisms and halophilic archaea has had an effect on the genome evolution of *H. boliviensis *as well (Figure 4). Yet, 9% of the proteins of *H. boliviensis *did not cluster with any of the proteins belonging to the reference microorganisms (Figure 4). They may form phylogenetic groups with proteins of microorganisms that were not included in these studies.

Under the neutral mutation-random drift theory, it is assumed that a certain fraction of new mutation are free of constraint or are selectively neutral, while the rest have deleterious effects and are selectively eliminated [35]. Nevertheless, Figures 1 and 4 imply that most mutations found in proteins related to carbohydrate transport and metabolism were a result of HGT, which agree on some criteria that point out that genetic drift is not sufficient for claiming neutrality [36], and on a resent observation that estimated that about 60% of the genome evolution of prokaryotes is dominated by HGT [2]. Furthermore, HGT can be related to adaptation of *H. boliviensis *to its environment (Figures 1, 2) and might, therefore, be selected to attain an optimum physiological response of the species to its habitat [3]. Yet, nearly neutral mutations could be inferred from Figure 3 and Additional file 1: Table S1, suggesting a continuous evolution of the proteins [1].

### Metabolic assimilation of carbohydrates by *H. boliviensis*

**Figure 5:**
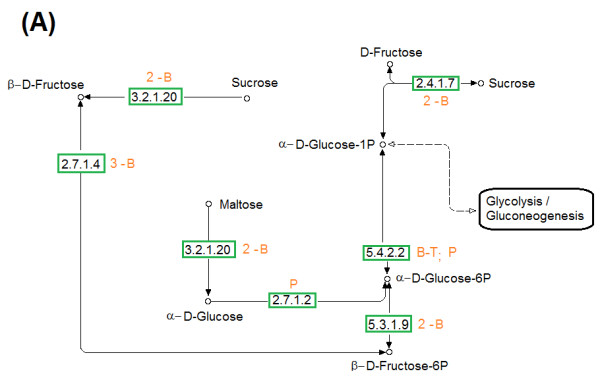
**Assimilation of carbohydrates in *H. boliviensis *by (A) The starch and sucrose metabolism**. Numbers and abbreviations for each metabolic step refer to the number of alleles and the cluster that the alleles formed with: P, Proteobacteria; B, bacteria; T, thermophilic archaea; HA, halophilic archaea; A, archaea (non halophilic and non thermophilic) and combinations of these groups of organisms; NC denotes that no cluster was formed between proteins of *H. boliviensis *and proteins belonging to the microorganisms used as reference. EC numbers for the enzymes in the metabolisms are pointed out as classified in the KEGG pathway database, and are listed in Additional file 3: Table S3 [37].

The metabolic routes in *H. boliviensis *for the assimilation of carbohydrates were obtained by matching the highest identities of enzymes derived from its genome with the KEGG pathway database [37]. The studies began searching for enzymes of *H. boliviensis *that form part of the starch and sucrose metabolism pathway. Although *H. boliviensis *is unable to hydrolyze starch [15], it assimilates maltose, glucose and other oligosaccharides obtained after the hydrolysis of starch [38]. Furthermore, *Halomonas boliviensis *can grow on sucrose [15]. We deduced in this study that both maltose and sucrose are metabolized via α-D-glucose-mono-phosphorylated before entering the glycolysis and gluconeogenesis pathway (Figure 5). Figure 5 shows further a high level of polymorphism for most of the enzymes in this metabolic route.

**Figure 6:**
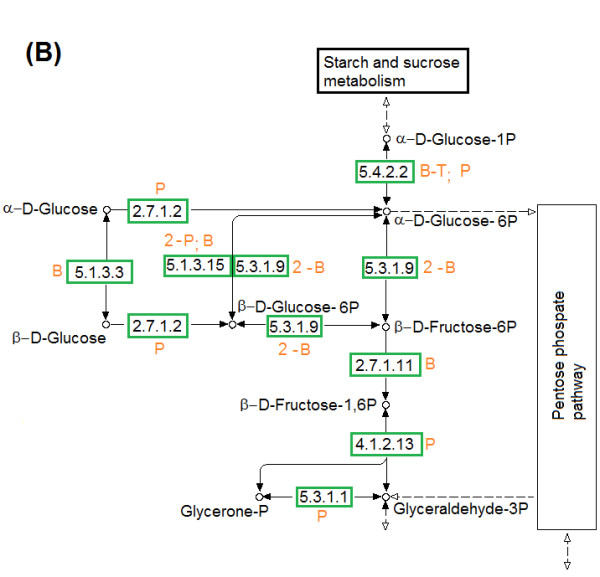
**Assimilation of carbohydrates in *H. boliviensis *by (B) The first steps of glycolysis and gluconeogenesis**. Name of the enzymes, numbers of alleles and abbreviations are referred as in Figure 5.

**Figure 7:**
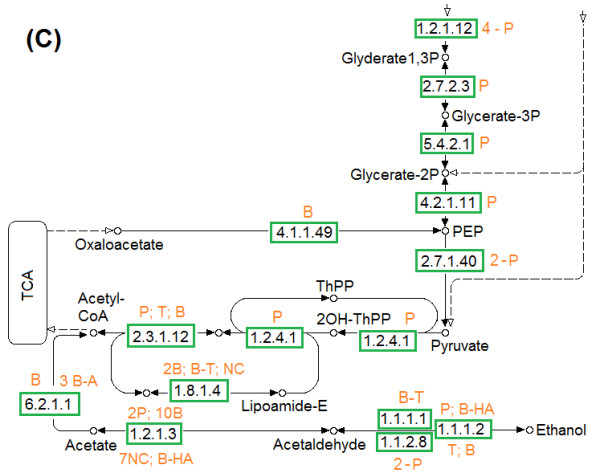
**Assimilation of carbohydrates in *H. boliviensis *by (C) The culmination steps of glycolysis and gluconeogenesis**. For the transformation of acetatyl CoA to ethanol, COGs for energy production and conversion and lipid transport and metabolism in cells were included. Name of the enzymes, numbers of alleles and abbreviations are referred as in Figure 5.

Similar polymorphism was observed in glycolysis and gluconeogenesis pathways of *H. boliviensis *(Figures 6, 7). For the first part of the metabolism, *H. boliviensis *obtained most of its gens by HGT among bacteria (Figure 6), whereas the enzymes at the bottom of the pathway were mainly related to enzymes of the Proteobacteria (Figure 7). Glycolysis in *H. boliviensis *concluded with the 2-oxoglutarate dehydrogenase complex (PDHC) (Figure 7), that is part of the pyruvate dehydrogenases family. This route is characterized by generation of NADH, and is commonly found in Gram-negative bacteria [39]. The enzymes that form part of PDHC in *H. boliviensis *were obtained in part from thermophilic archaea and mesophilic bacteria (Figure 7). Archaea do not utilize a pyruvate dehydrogenase complex to transform pyruvate to acetyl-CoA rather they accomplish the transformation using 2-oxoacid oxidoreductases [40]. However, putative enzyme sequences that form part of the PDHC can be found in the genome sequences of thermophilic [24,25] and halophilic archaea [24,25,41]. The protein divergence and gene duplication (Figure 3 and 7) may provide evidence of adaptive evolution of the metabolism [13]. The diversity of enzymes that can accomplish the same function in *H. boliviensis *explains its versatility in the assimilation of carbohydrates and other carbon sources (e.g. acetate and short chain fatty acids) [18,38]. The polymorphic metabolism of *H. boliviensis *might also lead to an efficient generation of energy (ATP) and the reducing agent NADH to correlate with its fast cell growth and its capability to metabolize different carbon sources to PHB [18,38]. Besides the synthesis of PHB, excess of NADH could potentially be oxidized by *H. boliviensis *via a fermentative route to allow the formation of ethanol (Figure 7).

### Relationship of the enzymes involved in glycolysis and gluconeogenesis among Prokaryotes

**Figure 8:**
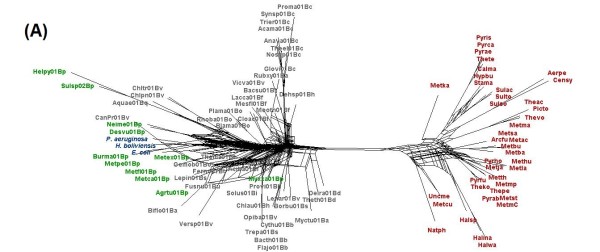
**Supernetworks constructed using the Split4tree program after combining phylogenetic trees of proteins of the glycolysis and gluconeogenesis metabolisms in *H. boliviensis *and reference strains**. The figures included combination of trees corresponding to COGs **(A) **0126, 0149 and 0837. Names of the archaeal and bacterial species corresponding to each abbreviation used in the figure are listed in Additional file 4: Table S4.

**Figure 9:**
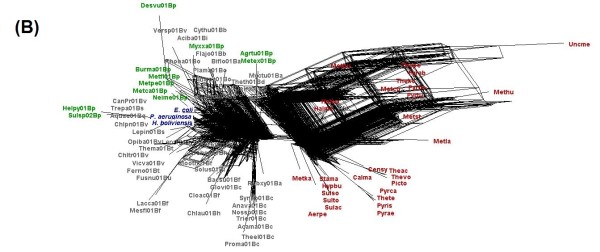
**Supernetworks constructed using the Split4tree program after combining phylogenetic trees of proteins of the glycolysis and gluconeogenesis metabolisms in *H. boliviensis *and reference strains**. The figures included combination of trees corresponding to COGs **(B) **0126, 0149, 0837, 0469, 0696 and 0837. Names of the archaeal and bacterial species corresponding to each abbreviation used in the figure are listed in Additional file 4: Table S4.

**Figure 10:**
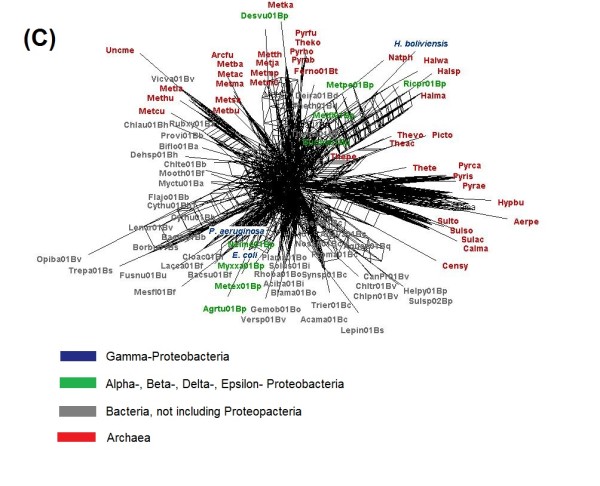
**Supernetworks constructed using the Split4tree program after combining phylogenetic trees of proteins of the glycolysis and gluconeogenesis metabolisms in *H. boliviensis *and reference strains**. The figures included combination of trees corresponding to COGs **(C) **0057, 0126, 0148, 0149, 0166, 0191, 0205, 0235, 0365, 0469, 0508, 0696, 0837, 1012, 1063, 1109, 1249, 1454, 1866, 2017, 2609 and 4993. Names of the archaeal and bacterial species corresponding to each abbreviation used in the figure are listed in Additional file 4: Table S4.

Considering the degree of polymorphism in the metabolic routes of *H. boliviensis*, we wonder whether this trend could be followed by other microorganisms in its environment. To address this question, supernetworks were constructed by combining the phylogenetic trees related to glycolysis and gluconeogenesis. Figure 8 shows a supernetwork obtained after combining three phylogenetic trees; two of them derived from COGs that are considered among the 102 genes that contain taxonomic information that discriminate well bacteria and archaea in already known families and genera [4]. The internetwork relationship among microorganisms shown in Figure 8 denotes that HGT occurred among bacteria, archaea and between bacteria and archaea. A supernetwork obtained from six trees reflected a higher effect of HGT among microorganisms (Figure 9). In Figure 9, taxonomic differentiation between proteins of bacteria and archaea was barely observed, although *H. boliviensis *was still clustered with other γ-Proteobacteria, i.e. *Escherichia coli *and *Pseudomonas aeruginosa*. Finally, 22 phylogenetic trees related to glycolysis and gluconeogenesis were used to attain a supernetwork (Figure 10). Taxonomic differentiation among the proteins of the microorganisms was no longer observed (Figure 10), hence suggesting that flow of genes involved in glycolysis and gluconeogenesis among Prokaryotes was significant. Experimental analysis demonstrated polymorphism for the enzymes that form part of glycolysis in *E. coli *[42], whereas various different *Halomonas *and *Chromohalobacter *species grow on several common carbon sources and are able to produce PHB [43]. On the whole, our studies imply that the availability of the selection of a particular carbohydrate by a microbial species should be related to the rate of evolution of the enzymes, and might be linked not only to the size of the microbial population, as stated by the nearly neutral theory of evolution [1], but also to the groups of microorganisms able to thrive in a particular niche.

### Use of combination of carbohydrates for the production of PHB by *H. boliviensis*

**Figure 11:**
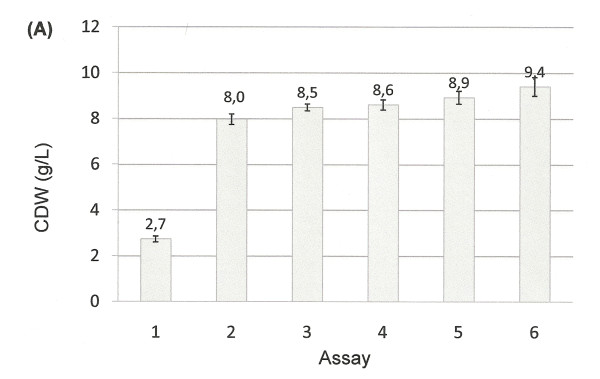
**Cell growth and PHB production by *H. boliviensis *using different combinations of carbohydrates and molasses**. **(A) **Cell dry weight (CDW) in batch culture using 1.5% (w/v) sucrose and 1% (w/v) glucose. Assay numbers refer to the concentration of carbohydrates added to the medium, %(w/v): 1) 2.5 sucrose, 2) 2.0 sucrose and 0.5 glucose, 3) 1.5 sucrose and 1 glucose, 4) 1.0 sucrose and 1.5 glucose, 5) 0.3 sucrose, 0.7 glucose and 1.5 dried molasses and 6) 2.5 dried molasses. All experiments were performed in shake flasks at 35°C and 220 rpm of agitation. All experiments were performed in triplicate. The error bars refer to the SD of the average values.

**Figure 12:**
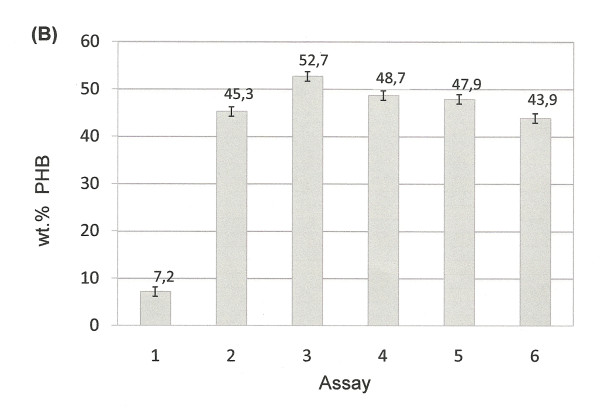
**Cell growth and PHB production by *H. boliviensis *using different combinations of carbohydrates and molasses**. **(B) **PHB accumulation in batch culture using 1.5% (w/v) sucrose and 1% (w/v) glucose. Assay numbers refer to the concentration of carbohydrates added to the medium, %(w/v): 1) 2.5 sucrose, 2) 2.0 sucrose and 0.5 glucose, 3) 1.5 sucrose and 1 glucose, 4) 1.0 sucrose and 1.5 glucose, 5) 0.3 sucrose, 0.7 glucose and 1.5 dried molasses and 6) 2.5 dried molasses. All experiments were performed in shake flasks at 35°C and 220 rpm of agitation. All experiments were performed in triplicate. The error bars refer to the SD of the average values.

The aforementioned results revealed that the rate of evolution, mutations and the molecular interaction between *H. boliviensis *and other microorganisms in its environment influenced significantly the evolution of the carbohydrate transport and metabolism in this bacterium--a similar evolutionary pattern might be expected in other prokaryotes. However, phenotypic traits concerning microbial growth on different carbon sources are stamps of different phylogenetic groups and species. We hypothesized that the amount and type of carbon sources in a particular environment should also influence the fitness of glycolysis and gluconeogenesis fluxes in *H. boliviensis*. The influence of the environment on the functional features of enzymes is a context not commonly evaluated in evolutionary theories of metabolic pathways [12,14]. For this reason, we decide to use various combinations of glucose and sucrose concentrations as precursors for PHB synthesis in *H. boliviensis *(Figures 11, 12).

Three alleles of the PHB synthases were found in the genome of *H. boliviensis *(Additional file 5: Figure S1). The three alleles are closely related to PHB synthases of Proteobacteria. Moreover, *H. boliviensis *A2 was clustered with two alleles of PHB polymerases of *Halomonas *sp. TD01 (Figure S1); one of these alleles (phaC1) was previously reported [6]. However, a third allele of *Halomonas *sp. TD01 (named phaC2) showed a distant phylogenetic relationship to the PHB synthases of Proteobacteria (Figure S1); phaC2 might have been acquired by HGT [6]. Research on the PHB polymerization and depolymerization pathways in *H. boliviensis *is in progress. PHB production by *H. boliviensis *was performed in shake flask experiments under nitrogen limitation conditions (i.e. a low concentration sodium glutamate was added to the culture medium to limit the cell growth). When sucrose was used as the sole carbon source, the accumulation of PHB in *H. boliviensis *(7.2 wt%) and cell growth (2.7 g/L) were low compared to those obtained with combinations of sucrose and glucose (Figures 11,12). Cell growth increased as the amount of glucose was higher in the medium to reach 8.6 g/L (Figure 11), while the maximum PHB content in *H. boliviensis *was 52.7 wt% when 1.5% (w/v) sucrose and 1.0% (w/v) glucose were included in the medium composition (Figure 12). The use of molasses enhanced to some extent the cell growth, c.a. 9.4 g/L, but the PHB accumulated in the cells was lower, 43.9 wt% (Figures 11, 12). Both the cell density and the maximum PHB yield attained by *H. boliviensis *are higher to those reported using glucose as carbon source, i.e. 5.3 g/L and 45 wt% respectively, under similar culture conditions [16].

**Figure 13:**
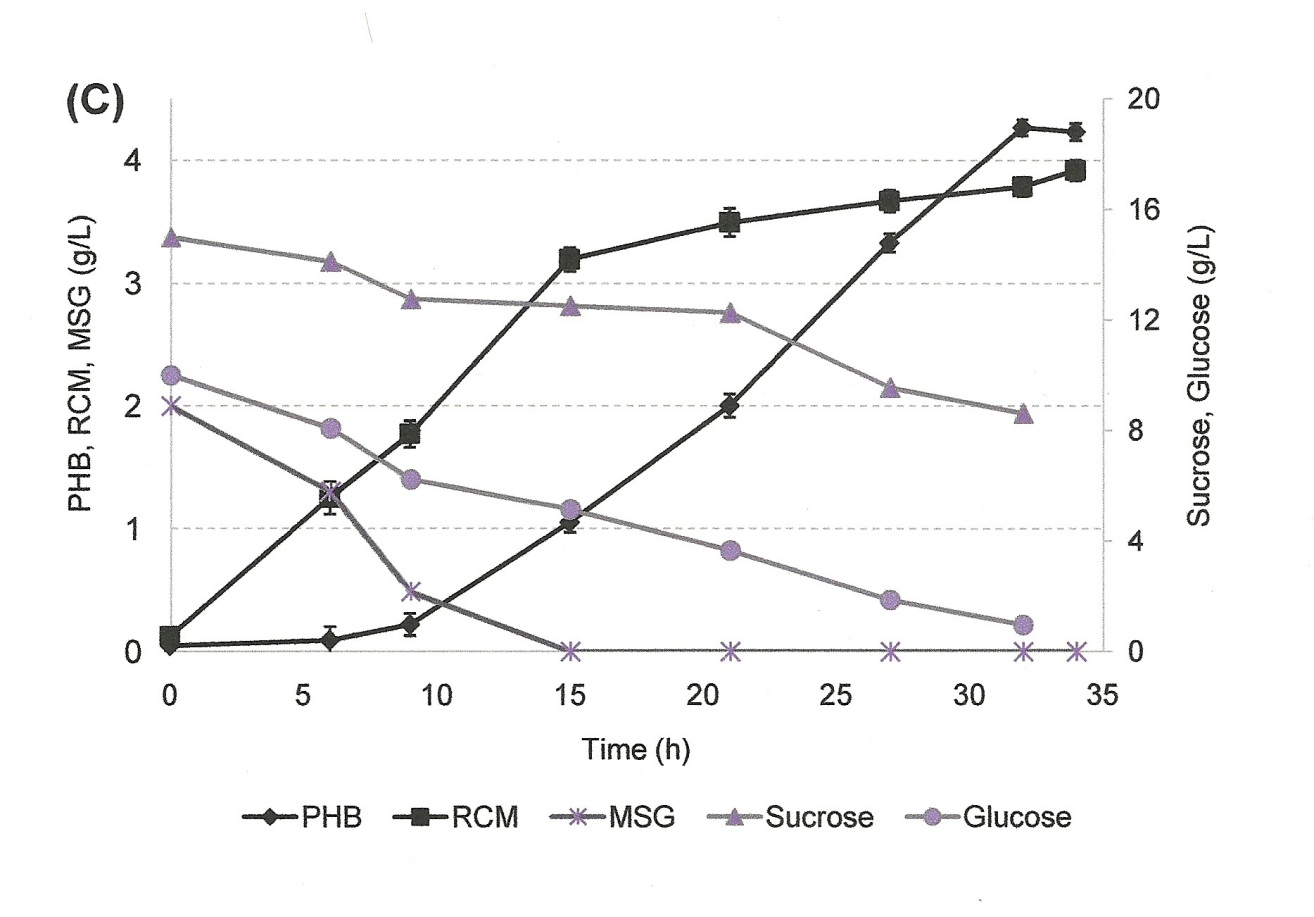
**Cell growth and PHB production by *H. boliviensis *using different combinations of carbohydrates and molasses**. **(C) **Carbohydrate assimilation and PHB production in batch culture using 1.5% (w/v) sucrose and 1% (w/v) glucose. All experiments were performed in shake flasks at 35°C and 220 rpm of agitation. The abbreviations RCM and MSG in the figure refer to residual cell mass and mono sodium glutamate, respectively. All experiments were performed in triplicate. The error bars refer to the SD of the average values.

Glucose and sucrose uptake and assimilation were analyzed further using the optimum sucrose and glucose ratio, i.e. 1.5:1, for PHB production (Figure 13). Residual cell mass (RCM), which is the cell biomass without the polymer inclusions, was used to analyze the active cell growth. Glucose and sucrose were assimilated parallelly during the exponential phase of growth of *H. boliviensis *(Figure 13). However, glucose consumption rate by *H. boliviensis *shows a linear decrease after 9 hours of cultivation when sodium glutamate was almost depleted from the medium and PHB synthesis was triggered (Figure 13). Sucrose concentration in the medium was constant from 9 to 21 h of cultivation and was only reduced when the concentration of glucose in the medium was low (Figure 13). Glucose generates a higher amount of energy and NADH in *H. boliviensis *than sucrose because the CDW and PHB reached by *H. boliviensis *using glucose were much higher than those achieved when sucrose was used as the sole carbon source (Figures 11, 12) [16]. Nevertheless, an overflow of NADH is known to obstruct the tricarboxylic acid cycle because of inhibition of citrate synthase [44]. An adequate ratio of glucose and sucrose in the medium promoted an appropriate balance of energy for an active cell growth (Figures 11, 13), albeit Figure 13 reflects that glucose is preferred during the PHB accumulation in *H. boliviensis *due to an excess of NADH in the cytoplasm of cells improves the polymer synthesis [19,44].

The maximum PHB concentration and volumetric productivity reached by *H. boliviensis *were 4.3 g/L and 0.13 g/L/h, respectively; they are comparable to those reached by *Cupriavidus necator*, i.e. 5.1 g/L and 0.11 g/L/h [45], and to those reported for a recombinant *E. coli *strain, c.a. 7.2 g/L and 0.15 g/L/h [46]. The medium for *C. necator *and *E. coli *contained glucose as the carbon source for experiments performed in shake flasks. Under similar culture conditions, *Azotobacter vinelandii *led to a PHB concentration of 7.5 g/L and a productivity of 0.30 g/L/h [47]. These bacteria attained among the highest productions of PHB, and are recognized for their potential utilization at industrial scales [19,21].

The viability of the commercialization of PHB is dependent upon the reduction of the total production costs [22]. The price of the carbon source supplied in the culture medium may account up to 40% of the total production costs [22]. Sucrose is at least two times cheaper than glucose while molasses are cheaper than sucrose. The results obtained for the production of PHB by *H. boliviensis *(Figures 11, 13) suggest that an agricultural surplus such as molasses could be used during the bioprocess scale up to stimulate the cell growth; furthermore an optimum ratio of sucrose and glucose should be added in the culture medium of the largest bioreactor used in a process to induce a high polymer production. Replacing partially glucose by sucrose and molasses should surly reduce the production costs of the polymer and lead also to an environmentally friendly bioprocess. Nevertheless, fed-batch cultivations systems are yet to be performed with *H. boliviensis *using combinations of carbohydrates to reveal their potential in a large scale process.

## Conclusions

The genome size and number of genes found in *H. boliviensis *were similar to those determined for other halophilic bacteria of the family *Halomonadaceae*. The ability of *H. boliviensis *to grow on different carbon sources is explained by the high number of genes related to the carbohydrate uptake and metabolism. Interestingly, most of these genes were obtained from other bacteria by HGT, only 34% of the genes evolved as proteins belonging to Proteobacteria, while 13% of the genes were transferred from haloarchaea and thermophilic archaea. Furthermore, the diversity of enzymes that have the same physiological function led to polymorphism in the metabolic routs. Results obtained in this article depict that most genetic modifications and enzyme polymorphism in the genome of *H. boliviensis *were mainly influenced by HGT rather than nearly neutral mutations. Molecular adaptation and evolution experienced by *H. boliviensis *were also a response to environmental conditions such as the type and amount of carbohydrates in its ecological niche. Consequently, the genome evolution of *H. boliviensis *showed to be strongly influenced by the type of microorganisms, genetic interaction among microbial species and its environment. Such trend should also be experienced by other prokaryotes. A system for PHB production by *H. boliviensis *that takes into account the evolutionary adaptation of this bacterium to the assimilation of combinations of carbohydrates suggests the feasibility of a bioprocess economically viable and environmentally friendly.

## Competing interests

The authors declare that they have no competing interests.

## Authors' contributions

DG, AB-S, CC-O, MG-M and JQ performed the evolutionary analyses on the COGs corresponding to the carbohydrate transport and metabolism of *H. boliviensis*. JQ constructed the supernetworks. JQ, DG and NC-Q studied the PHB production by *H. boliviensis*. All authors wrote the manuscript and approved the final version.

## *Halomonas boliviensis *genome sequence

This whole genome shotgun project was deposited at DDBJ, EMBL and GenBank under the accession number AGQZ00000000. The version described in this paper is the first version, AGQZ01000000.

## Supplementary Material

Additional file 1 **Table S1**. Clusters of orthologous genes (COGs) of *H. boliviensis *related to carbon transport and metabolism. Click here for file

Additional file 2 **Table S2**. List of GenBank accession numbers for the microorganisms shown in the phylogenetic trees in Figure 1, 2, 3. Click here for file

Additional file 3 **Table S3**. EC numbers and COG classification of the enzymes involved in starch metabolism, glycolysis and gluconeogenesis in *H. boliviensis*. Click here for file

Additional file 4 **Table S4**. List of species and abbreviations of the 100 microorganisms (59 Bacteria and 41 Archaea) used as reference. Abbreviations are named as described by Puigbò, et al. 2009. Click here for file

Additional file 5 **Figure S1**. Click here for file
