# Supplementary material for: Evolutionary patterns of carbohydrate transport and metabolism in Halomonas boliviensis as derived from its genome sequence: influences on polyester production
Source: Aquat Biosyst. 2012 Apr 17;8:9. doi: 10.1186/2046-9063-8-9 (PMC3384467; doi:10.1186/2046-9063-8-9)
Supplement: Additional file 1 — Table S1. Clusters of orthologous genes (COGs) of H. boliviensis related to carbon transport and metabolism. [file 2046-9063-8-9-S1.DOC]

**TABLE S1.** Clusters of orthologous genes (COGs) of *H. boliviensis* related to carbon transport and metabolism.

‡ Refers to the number of alleles and the cluster that the alleles formed with: P, proteobacteria; B, bacteria; T, thermophilic archaea; HA, halophilic archaea; combinations of these groups of organisms and NC denotes that no cluster was formed.

| **COG** | **Protein function** | **Number of alleles** | **Clusters formed by the alleles^‡^** |
| --- | --- | --- | --- |
| 21 | Transketolase | 1 | P |
| 33 | Phosphoglucomutase | 1 | B |
| 36 | Pentose-5-phosphate-3-epimerase | 1 | P |
| 57 | Glyceraldehyde-3-phosphate dehydrogenase / erythrose-4-phosphate dehydrogenase | 4 | 4 P |
| 61 | Predicted sugar kinase | 1 | P |
| 63 | Predicted sugar kinase | 1 | B |
| 120 | Ribose 5-phosphate isomerase | 1 | P |
| 126 | 3-phosphoglycerate kinase | 1 | P |
| 148 | Enolase | 1 | P |
| 149 | Triosephosphate isomerase | 1 | P |
| 166 | Glucose-6-phosphate isomerase | 2 | 2 - B |
| 176 | Transaldolase | 1 | P |
| 191 | Fructose/tagatose bisphosphate aldolase | 1 | P |
| 205 | 6-phosphofructokinase | 1 | B |
| 235 | Ribulose-5-phosphate 4-epimerase and related epimerases and aldolases | 3 | 2 - P  1 - B |
| 246 | Mannitol-1-phosphate/altronate dehydrogenases | 2 | 1 - P  1 - NC |
| 279 | Phosphoheptose isomerase | 2 | 1 - P  1 - B |
| 363 | 6-phosphogluconolactonase/Glucosamine-6-phosphate isomerase/deaminase | 1 | P |
| 364 | Glucose-6-phosphate 1-dehydrogenase | 1 | P |
| 366 | Glycosidases | 2 | 2 - B |
| 395 | ABC-type sugar transport system, permease component | 7 | 1 - P  4 - B  1 - B, HA  1 - B, T |
| 406 | Fructose-2,6-bisphosphatase | 3 | 1 - P  2 - B |
| 469 | Pyruvate kinase | 2 | 2 - P |
| 483 | Archaeal fructose-1,6-bisphosphatase and related enzymes of inositol monophosphatase family | 4 | 1 - P  1 - B  2 - B, HA |
| 524 | Sugar kinases, ribokinase family | 3 | 3 - B |
| 574 | Phosphoenolpyruvate synthase/pyruvate phosphate dikinase | 1 | P |
| 662 | Mannose-6-phosphate isomerase | 1 | B |
| 676 | Uncharacterized enzymes related to aldose 1-epimerase | 1 | B |
| 696 | Phosphoglyceromutase | 1 | P |
| 726 | Predicted xylanase/chitin deacetylase | 2 | 1 - P  1 - B |
| 800 | 2-keto-3-deoxy-6-phosphogluconate aldolase | 2 | 1 - P  1 - HA |
| 837 | Glucokinase | 1 | P |
| 1015 | Phosphopentomutase | 1 | P |
| 1070 | Sugar (pentulose and hexulose) kinases | 2 | 2 - B |
| 1080 | Phosphoenolpyruvate-protein kinase (PTS system EI component in bacteria) | 1 | B |
| 1082 | Sugar phosphate isomerases/epimerases | 2 | 2 - B |
| 1105 | Fructose-1-phosphate kinase and related fructose-6-phosphate kinase (PfkB) | 1 | B |
| 1109 | Phosphomannomutase | 2 | 1 - P  1 - B, T |
| 1129 | ABC-type sugar transport system, ATPase component | 3 | 1 - P  1 - B  1 - B, T |
| 1172 | Ribose/xylose/arabinose/galactoside ABC-type transport systems, permease components | 3 | 2 - B  1 - T |
| 1175 | ABC-type sugar transport systems, permease components | 7 | 1 - B  4 - B, T  2 - B, HA |
| 1299 | Phosphotransferase system, fructose-specific IIC component | 1 | P |
| 1312 | D-mannonate dehydratase | 1 | B |
| 1363 | Cellulase M and related proteins | 1 | B |
| 1472 | Beta-glucosidase-related glycosidases | 1 | P |
| 1593 | TRAP-type C4-dicarboxylate transport system, large permease component | 15 | 6 - P  5 - B  1 - B, HA  3 - NC |
| 1638 | TRAP-type C4-dicarboxylate transport system, periplasmic component | 17 | 6 - P  5 - B  6 - NC |
| 1653 | ABC-type sugar transport system, periplasmic component | 7 | 3 - P  3 - B  1 - T |
| 1803 | Methylglyoxal synthase | 1 | B |
| 1830 | DhnA-type fructose-1,6-bisphosphate aldolase and related enzymes | 1 | B |
| 1850 | Ribulose 1,5-bisphosphate carboxylase, large subunit | 1 | B |
| 1879 | ABC-type sugar transport system, periplasmic component | 3 | 2 - B  1 - B, T |
| 1929 | Glycerate kinase | 1 | B |
| 2017 | Galactose mutarotase and related enzymes | 1 | B |
| 2133 | Glucose/sorbosone dehydrogenases | 1 | HA |
| 2513 | PEP phosphonomutase and related enzymes | 1 | P |
| 2721 | Altronate dehydratase | 1 | B |
| 2814 | Arabinose efflux permease | 4 | 4 - NC |
| 2956 | Predicted N-acetylglucosaminyl transferase | 1 | P |
| 3090 | TRAP-type C4-dicarboxylate transport system, small permease component | 11 | 2 - P  6 - B  2 - B, HA  1 - NC |
| 3265 | Gluconate kinase | 1 | B |
| 3386 | Gluconolactonase | 1 | B |
| 3459 | Cellobiose phosphorylase | 1 | B |
| 3693 | Beta-1,4-xylanase | 1 | B |
| 3717 | 5-keto 4-deoxyuronate isomerase | 1 | B |
| 3718 | Uncharacterized enzyme involved in inositol metabolism | 1 | B |
| 3734 | 2-keto-3-deoxy-galactonokinase | 1 | B |
| 3839 | ABC-type sugar transport systems, ATPase components | 9 | 1 - P  6 - B  1 - T  1 - B, T |
| 3936 | Protein involved in polysaccharide intercellular adhesin (PIA) synthesis/biofilm formation | 1 | B |
| 4678 | Muramidase (phage lambda lysozyme) | 1 | P |
| 4993 | Glucose dehydrogenase | 2 | 2 - P |
