# Supplementary material for: Evolutionary patterns of carbohydrate transport and metabolism in Halomonas boliviensis as derived from its genome sequence: influences on polyester production
Source: Aquat Biosyst. 2012 Apr 17;8:9. doi: 10.1186/2046-9063-8-9 (PMC3384467; doi:10.1186/2046-9063-8-9)
Supplement: Additional file 2 — Table S2. List of GenBank accession numbers for the microorganisms shown in the phylogenetic trees in Figure 1, 2, 3. [file 2046-9063-8-9-S2.DOC]

**Table S2.** List of GenBank accession numbers for the microorganisms shown in the phylogenetic trees in Figures 1, 2 and 3.

| **Microorganism** | **Fig. 1** | **Fig. 2** | **Fig. 3** |
| --- | --- | --- | --- |
| *Aeropyrum pernix* | - | - | NP147274 |
| *Hyperthermus butylicus* | - | - | YP001013177 |
| *Staphylothermus marinus* | - | - | YP001040907 |
| *Sulfolobus acidocaldarius* | YP256707 | - | - |
| *Sulfolobus solfataricus* | - | - | NP342465 |
| *Sulfolobus tokodaii* | - | - | NP377586 |
| *Caldivirga maquilingensis* | - | - | YP001540411 |
| *Pyrobaculum aerophilum* | - | - | NP558871 |
| *Pyrobaculum calidifontis* | - | - | YP001054935 |
| *Pyrobaculum islandicum* | - | - | YP930620 |
| *Thermofilum pendens* | [YP920610](http://www.ncbi.nlm.nih.gov/protein/119720115?report=genbank&log$=prottop&blast_rank=1&RID=MTVJRZSP012) | - | [YP919742](http://www.ncbi.nlm.nih.gov/protein/119719247?report=genbank&log$=prottop&blast_rank=1&RID=MWF36TTX01N) |
| *Thermoproteus tenax* | - | - | YP004893586 |
| *Haloarcula marismortui* | - | YP137536 | - |
| *Haloquadratum walsbyi* | - | YP657267 | - |
| *Methanococcus jannaschii* | - | - | NP247072 |
| *Methanococcus maripaludis* | - | - | YP001098314 |
| *Methanococcus maripaludis* | - | - | NP988725 |
| *Methanoculleus marisnigri* | - | - | YP001046442 |
| *Methanospirillum hungatei* | - | - | YP501946 |
| *Methanococcoides burtonii* | - | - | YP565204 |
| *Methanosarcina acetivorans* | - | - | NP618761 |
| *Methanosarcina barkeri fusaro* | - | - | YP303740 |
| *Methanosarcina mazei* | - | - | NP632739 |
| *Pyrococcus abyssi* | - | - | NP127086 |
| *Pyrococcus furiosus* | - | - | NP578917 |
| *Pyrococcus horikoshii* | - | - | NP142537 |
| *Thermococcus kodakaraensis* | - | - | YP182924 |
| *Picrophilus torridus* | - | - | YP023114 |
| *Thermoplasma acidophilum* | - | - | NP394355 |
| *Thermoplasma volcanium* | - | - | NP111539 |
| *Acidobacteria bacterium* | - | - | YP593743 |
| *Solibacter usitatus* | YP829009 | YP826524 | YP826243 |
| *Mycobacterium tuberculosis* | - | - | NP216133 |
| *Bifidobacterium longum* | NP695266 | - | NP696160 |
| *Rubrobacter xylanophilus* | YP643726 | YP642872 | - |
| *Bacteroides thetaiotaomicron* | - | NP809402 | NP811753 |
| *Flavobacterium johnsoniae* | - | YP001196587 | YP001193493 |
| *Cytophaga hutchinsonii* | - | - | YP678011 |
| *Chlamydia trachomatis* | - | - | NP219839 |
| *Chlamydophila pneumoniae* | - | - | NP224305 |
| *Chloroflexus aurantiacus* | YP001635886 | - | YP001636715 |
| *Synechocystis sp* | - | NP442562 | NP442551 |
| *Thermosynechococcus elongatus* | - | NP682718 | NP681306 |
| *Gloeobacter violaceus* | NP924676 | NP927217 | NP926269 |
| *Anabaena variabilis* | - | YP322958 | YP321013 |
| *Nostoc sp* | - | NP488811 | NP486604 |
| *Trichodesmium erythraeum* | - | YP724336 | - |
| *Prochlorococcus marinus subsp marinus* | - | NP874648 | - |
| *Acaryochloris marina* | - | YP001519178 | YP001515306 |
| *Deinococcus radiodurans* | - | - | NP296354 |
| *Thermus thermophilus* | - | YP006013 | YP005580 |
| *Bacillus subtilis subsp subtilis* | BAI87238 | NP390092 | - |
| *Clostridium acetobutylicum* | - | NP349575 | - |
| *Mesoplasma florum* | - | - | YP053416 |
| *Lactobacillus casei* | YP805611 | YP001988771 | - |
| *Moorella thermoacetica* | YP429487 | YP429294 | - |
| *Fusobacterium nucleatum subsp nucleatum* | NP602696 | - | - |
| *Lentisphaera araneosa* | ZP01874250 | ZP01875894 | ZP01873326 |
| *Victivallis vadensis* | - | ZP06244826 | ZP06242649 |
| *Blastopirellula marina* | ZP01093469 | ZP01091408 | ZP01091188 |
| *Gemmata obscuriglobus* | ZP02733555 | ZP02732644 | ZP02734836 |
| *Planctomyces maris* | ZP01853600 | ZP01856549 | ZP01853612 |
| *Rhodopirellula baltica* | NP865544 | - | NP869344 |
| *Agrobacterium tumefaciens* | NP356332 | NP356163 | NP356860 |
| *Methylobacterium extorquens* | - | YP001639476 | YP001640206 |
| *Burkholderia mallei* | ZP02447535 | YP109525 | YP107422 |
| *Methylibium petroleiphilum* | - | - | YP001019487 |
| *Methylobacillus flagellatus* | - | YP544869 | YP546352 |
| *Neisseria meningitidis* | - | NP274408 | NP273151 |
| *Desulfovibrio vulgaris subsp vulgaris* | - | - | YP011726 |
| *Myxococcus xanthus* | - | - | YP634424 |
| *Helicobacter pylori* | - | NP207890 | - |
| *Sulfurovum sp* | - | - | YP001357753 |
| *Escherichia coli* | EFZ67937 | NP288287 | NP416368 |
| *Methylococcus capsulatus* | - | YP112578 | YP115002 |
| *Pseudomonas aeruginosa* | NP250638 | NP251821 | NP250189 |
| *Borrelia burgdorferi* | - | - | NP212482 |
| *Leptospira interrogans serovar Copenhageni* | - | - | NP714897 |
| *Treponema pallidum subsp pallidum* | - | NP219007 | - |
| *Fervidobacterium nodosum* | YP001409577 | - | - |
| *Thermotoga maritima* | NP228763 | NP227882 | NP228023 |
| *Opitutaceae bacterium* | - | - | ZP09661392 |
| *Methylokorus infernorum* | - | - | YP001938741 |
| *Halomonas boliviensis A1* | ZP09188000 | ZP09188472 | ZP09189203 |
| *Halomonas boliviensis A2* | ZP09188258 | ZP09188475 | ZP09188486 |
| *Halomonas boliviensis A3* | ZP09188252 | - | - |
