# Supplementary material for: Evolutionary patterns of carbohydrate transport and metabolism in Halomonas boliviensis as derived from its genome sequence: influences on polyester production
Source: Aquat Biosyst. 2012 Apr 17;8:9. doi: 10.1186/2046-9063-8-9 (PMC3384467; doi:10.1186/2046-9063-8-9)
Supplement: Additional file 3 — Table S3. EC numbers and COG classification of the enzymes involved in starch metabolism, glycolysis and gluconeogenesis in H. boliviensis. [file 2046-9063-8-9-S3.DOC]

**Table S3.** EC numbers and COG classification of the enzymes involved in starch metabolism, glycolysis and gluconeogenesis in *H. boliviensis*.

*Enzymes showed in Figure 5*

| **Enzyme** | **EC Number** | **COG** |
| --- | --- | --- |
| Alpha-glucosidase | 2.4.1.7 | COG0366 |
| Phosphomannomutase | 5.4.2.2 | COG1109 |
| Glucose-6-phosphate isomerase | 5.3.1.9 | COG0166 |
| Glucokinase | 2.7.1.2 | COG0837 |
| Alpha-glucosidase | 3.2.1.20 | COG0366 |
| Fructokinase | 2.7.1.4 | COG0524 |

*Enzymes showed in Figure 6*

| **Enzyme** | **EC Number** | **COG** |
| --- | --- | --- |
| Phosphoglucomutase | 5.4.2.2 | COG1109 |
| Glucose-6-phosphate isomerase | 5.3.1.9 | COG0166 |
| Glucokinase | 2.7.1.2 | COG0837 |
| Aldose 1-epimerase | 5.1.3.3 | COG2017 |
| Glucose-6-phosphate 1-epimerase | 5.1.3.15 | COG0235 |
| 6-Phosphofructokinase | 2.7.1.11 | COG0205 |
| Fructose-bisphosphate aldolase, class II | 4.1.2.13 | COG0191 |
| Triosephosphate isomerase (TIM) | 5.3.1.1 | COG0149 |

*Enzymes showed in Figure 7*

| **Enzyme** | **EC Number** | **COG** |
| --- | --- | --- |
| Glyceraldehyde 3-phosphate dehydrogenase | 1.2.1.12 | COG0057 |
| Phosphoglycerate kinase | 2.7.2.3 | COG0126 |
| Phosphoglycerate mutase | 5.4.2.1 | COG0696 |
| Enolase | 4.2.1.11 | COG0148 |
| Phosphoenolpyruvate carboxykinase (ATP) | 4.1.1.49 | COG1866 |
| Pyruvate kinase | 2.7.1.40 | COG0469 |
| Pyruvate dehydrogenase E1 component | 1.2.4.1 | COG2609 |
| Pyruvate dehydrogenase E2 component (dihydrolipoamide acetyltransferase) | 2.3.1.12 | COG0508 |
| Dihydrolipoamide dehydrogenase | 1.8.1.4 | COG1249 |
| Acetyl-CoA synthetase | 6.2.1.1 | COG0365 |
| Aldehyde dehydrogenase (NAD+) | 1.2.1.3 | COG1012 |
| Alcohol dehydrogenase | 1.1.1.1 | COG1454 |
| Alcohol dehydrogenase (NADP+) | 1.1.1.2 | COG1063 |
| Alcohol dehydrogenase (cytochrome c) | 1.1.2.8 | COG4993 |
