# Supplementary material for: Evolutionary patterns of carbohydrate transport and metabolism in Halomonas boliviensis as derived from its genome sequence: influences on polyester production
Source: Aquat Biosyst. 2012 Apr 17;8:9. doi: 10.1186/2046-9063-8-9 (PMC3384467; doi:10.1186/2046-9063-8-9)
Supplement: Additional file 4 — Table S4. List of species and abbreviations of the 100 microorganisms (59 Bacteria and 41 Archaea) used as reference. Abbreviations are named as described by Puigbò, et al. 2009. [file 2046-9063-8-9-S4.DOC]

**Table S4.** List of species and abbreviations of the 100 microorganisms (59 Bacteria and 41 Archaea) used as reference. Abbreviations are named as described by Puigbò, *et al.* 2009.

| **Abbreviation** | **Genus and species** | **Domain** | **Group** |
| --- | --- | --- | --- |
| Censy | *Cenarchaeum symbiosum* | Archaea | Crenarchaeota |
| Aerpe | *Aeropyrum pernix* | Archaea | Crenarchaeota |
| Hypbu | *Hyperthermus butylicus* | Archaea | Crenarchaeota |
| Stama | *Staphylothermus marinus F1* | Archaea | Crenarchaeota |
| Sulac | *Sulfolobus acidocaldarius DSM 639* | Archaea | Crenarchaeota |
| Sulso | *Sulfolobus solfataricus* | Archaea | Crenarchaeota |
| Sulto | *Sulfolobus tokodaii* | Archaea | Crenarchaeota |
| Calma | *Caldivirga maquilingensis IC-167* | Archaea | Crenarchaeota |
| Pyrae | *Pyrobaculum aerophilum* | Archaea | Crenarchaeota |
| Pyrca | *Pyrobaculum calidifontis JCM 11548* | Archaea | Crenarchaeota |
| Pyris | *Pyrobaculum islandicum DSM 4184* | Archaea | Crenarchaeota |
| Thepe | *Thermofilum pendens Hrk 5* | Archaea | Crenarchaeota |
| Thete | *Thermoproteus tenax* | Archaea | Crenarchaeota |
|  |  |  |  |
| Uncme | *Uncultured methanogenic archaeon* | Archaea | Euryarchaeota |
| Arcfu | *Archaeoglobus fulgidus* | Archaea | Euryarchaeota |
| Halma | *Haloarcula marismortui ATCC 43049* | Archaea | Euryarchaeota |
| Halsp | *Halobacterium sp* | Archaea | Euryarchaeota |
| Halwa | *Haloquadratum walsbyi* | Archaea | Euryarchaeota |
| Natph | *Natronomonas pharaonis* | Archaea | Euryarchaeota |
| Metth | *Methanobacterium thermoautotrophicum* | Archaea | Euryarchaeota |
| Metst | *Methanosphaera stadtmanae* | Archaea | Euryarchaeota |
| Metja | *Methanococcus jannaschii* | Archaea | Euryarchaeota |
| MetmC | *Methanococcus maripaludis C5* | Archaea | Euryarchaeota |
| Metmp | *Methanococcus maripaludis S2* | Archaea | Euryarchaeota |
| Metla | *Methanocorpusculum labreanum Z* | Archaea | Euryarchaeota |
| Metcu | *Methanoculleus marisnigri JR1* | Archaea | Euryarchaeota |
| Methu | *Methanospirillum hungatei JF-1* | Archaea | Euryarchaeota |
| Metka | *Methanopyrus kandleri* | Archaea | Euryarchaeota |
| Metbu | *Methanococcoides burtonii DSM 6242* | Archaea | Euryarchaeota |
| Metsa | *Methanosaeta thermophila PT* | Archaea | Euryarchaeota |
| Metac | *Methanosarcina acetivorans* | Archaea | Euryarchaeota |
| Metba | *Methanosarcina barkeri fusaro* | Archaea | Euryarchaeota |
| Metma | *Methanosarcina mazei* | Archaea | Euryarchaeota |
| Pyrab | *Pyrococcus abyssi* | Archaea | Euryarchaeota |
| Pyrfu | *Pyrococcus furiosus* | Archaea | Euryarchaeota |
| Pyrho | *Pyrococcus horikoshii* | Archaea | Euryarchaeota |
| Theko | *Thermococcus kodakaraensis KOD1* | Archaea | Euryarchaeota |
| Picto | *Picrophilus torridus DSM 9790* | Archaea | Euryarchaeota |
| Theac | *Thermoplasma acidophilum* | Archaea | Euryarchaeota |
| Thevo | *Thermoplasma volcanium* | Archaea | Euryarchaeota |
|  |  |  |  |
| Naneq | *Nanoarchaeum equitans* | Archaea | Nanoarchaeota |
|  |  |  |  |
| Aciba01Bi | *Acidobacteria bacterium Ellin345* | Bacteria | Acidobacteria |
| Solus01Bi | *Solibacter usitatus Ellin6076* | Bacteria | Acidobacteria |
|  |  |  |  |
| Myctu01Ba | *Mycobacterium tuberculosis H37Rv* | Bacteria | Actinobacteria |
| Biflo01Ba | *Bifidobacterium longum NCC2705* | Bacteria | Actinobacteria |
| Rubxy01Ba | *Rubrobacter xylanophilus DSM 9941* | Bacteria | Actinobacteria |
|  |  |  |  |
| Aquae01Bq | *Aquifex aeolicus VF5* | Bacteria | Aquificae |
|  |  |  |  |
| Bacth01Bb | *Bacteroides thetaiotaomicron VPI-5482* | Bacteria | Bacteroidetes |
| Flajo01Bb | *Flavobacterium johnsoniae UW101* | Bacteria | Bacteroidetes |
| Cythu01Bb | *Cytophaga hutchinsonii ATCC 33406* | Bacteria | Bacteroidetes |
|  |  |  |  |
| CanPr01Bv | *Candidatus Protochlamydia amoebophila UWE25* | Bacteria | Chlamydiae |
| Chltr01Bv | *Chlamydia trachomatis D/UW-3/CX* | Bacteria | Chlamydiae |
| Chlpn01Bv | *Chlamydophila pneumoniae AR39* | Bacteria | Chlamydiae |
|  |  |  |  |
| Chlte01Bb | *Chlorobium tepidum TLS* | Bacteria | Chlorobi |
| Provi01Bb | *Prosthecochloris vibrioformis DSM 265* | Bacteria | Chlorobi |
|  |  |  |  |
| Chlau01Bh | *Chloroflexus aurantiacus J-10-fl* | Bacteria | Chloroflexi |
| Dehsp01Bh | *Dehalococcoides sp BAV1* | Bacteria | Chloroflexi |
|  |  |  |  |
| Synsp01Bc | *Synechocystis sp PCC 6803* | Bacteria | Cyanobacteria |
| Theel01Bc | *Thermosynechococcus elongatus BP-1* | Bacteria | Cyanobacteria |
| Glovi01Bc | *Gloeobacter violaceus PCC 7421* | Bacteria | Cyanobacteria |
| Anava01Bc | *Anabaena variabilis ATCC 29413* | Bacteria | Cyanobacteria |
| Nossp01Bc | *Nostoc sp PCC 7120* | Bacteria | Cyanobacteria |
| Trier01Bc | *Trichodesmium erythraeum IMS101* | Bacteria | Cyanobacteria |
| Proma01Bc | *Prochlorococcus marinus subsp marinus str CCMP1375* | Bacteria | Cyanobacteria |
| Acama01Bc | *Acaryochloris marina MBIC11017* | Bacteria | Cyanobacteria |
|  |  |  |  |
| Deira01Bd | *Deinococcus radiodurans R1* | Bacteria | Deinococci |
| Theth01Bd | *Thermus thermophilus HB27* | Bacteria | Deinococci |
|  |  |  |  |
| Bacsu01Bf | *Bacillus subtilis subsp subtilis str 168* | Bacteria | Firmicutes |
| Cloac01Bf | *Clostridium acetobutylicum ATCC 824* | Bacteria | Firmicutes |
| Mesfl01Bf | *Mesoplasma florum L1* | Bacteria | Firmicutes |
| Lacca01Bf | *Lactobacillus casei ATCC 334* | Bacteria | Firmicutes |
| Mooth01Bf | *Moorella thermoacetica ATCC 39073* | Bacteria | Firmicutes |
|  |  |  |  |
| Fusnu01Bu | *Fusobacterium nucleatum subsp nucleatum ATCC 25586* | Bacteria | Fusobacteria |
|  |  |  |  |
| Lenar01Bv | *Lentisphaera araneosa HTCC2155* | Bacteria | Lentisphaerae |
| Vicva01Bv | *Victivallis vadensis ATCC BAA-548* | Bacteria | Lentisphaerae |
|  |  |  |  |
| Blama01Bo | *Blastopirellula marina DSM 3645* | Bacteria | Planctomycetes |
| Gemob01Bo | *Gemmata obscuriglobus* | Bacteria | Planctomycetes |
| Plama01Bo | *Planctomyces maris DSM 8797* | Bacteria | Planctomycetes |
| Rhoba01Bo | *Rhodopirellula baltica SH 1* | Bacteria | Planctomycetes |
|  |  |  |  |
| Agrtu01Bp | *Agrobacterium tumefaciens str C58* | Bacteria | Proteobacteria-Alpha |
| Metex01Bp | *Methylobacterium extorquens PA1* | Bacteria | Proteobacteria-Alpha |
| Ricpr01Bp | *Rickettsia prowazekii str Madrid E* | Bacteria | Proteobacteria-Alpha |
|  |  |  |  |
| Burma01Bp | *Burkholderia mallei ATCC 23344* | Bacteria | Proteobacteria-Beta |
| Metpe01Bp | *Methylibium petroleiphilum PM1* | Bacteria | Proteobacteria-Beta |
| Metfl01Bp | *Methylobacillus flagellatus KT* | Bacteria | Proteobacteria-Beta |
| Neime01Bp | *Neisseria meningitidis MC58* | Bacteria | Proteobacteria-Beta |
|  |  |  |  |
| Desvu01Bp | *Desulfovibrio vulgaris subsp vulgaris str Hildenborough* | Bacteria | Proteobacteria-Delta |
| Myxxa01Bp | *Myxococcus xanthus DK 1622* | Bacteria | Proteobacteria-Delta |
|  |  |  |  |
| Helpy01Bp | *Helicobacter pylori 26695* | Bacteria | Proteobacteria-Epsilon |
| Sulsp02Bp | *Sulfurovum sp NBC37-1* | Bacteria | Proteobacteria-Epsilon |
|  |  |  |  |
| Escco01Bp | *Escherichia coli K12* | Bacteria | Proteobacteria-Gamma |
| Metca01Bp | *Methylococcus capsulatus str Bath* | Bacteria | Proteobacteria-Gamma |
| Pseae01Bp | *Pseudomonas aeruginosa PAO1* | Bacteria | Proteobacteria-Gamma |
|  |  |  |  |
| Borbu01Bs | *Borrelia burgdorferi B31* | Bacteria | Spirochaetes |
| Lepin01Bs | *Leptospira interrogans serovar Copenhageni str Fiocruz L1-130* | Bacteria | Spirochaetes |
| Trepa01Bs | *Treponema pallidum subsp pallidum str Nichols* | Bacteria | Spirochaetes |
|  |  |  |  |
| Ferno01Bt | *Fervidobacterium nodosum Rt17-B1* | Bacteria | Thermotogae |
| Thema01Bt | *Thermotoga maritima MSB8* | Bacteria | Thermotogae |
|  |  |  |  |
| Opiba01Bv | *Opitutaceae bacterium TAV2* | Bacteria | Verrucomicrobia |
| Versp01Bv | *Methylokorus infernorum V4* | Bacteria | Verrucomicrobia |
