# Supplementary figures and images for: Evolutionary patterns of carbohydrate transport and metabolism in Halomonas boliviensis as derived from its genome sequence: influences on polyester production
Source: Aquat Biosyst. 2012 Apr 17;8:9. doi: 10.1186/2046-9063-8-9 (PMC3384467; doi:10.1186/2046-9063-8-9)

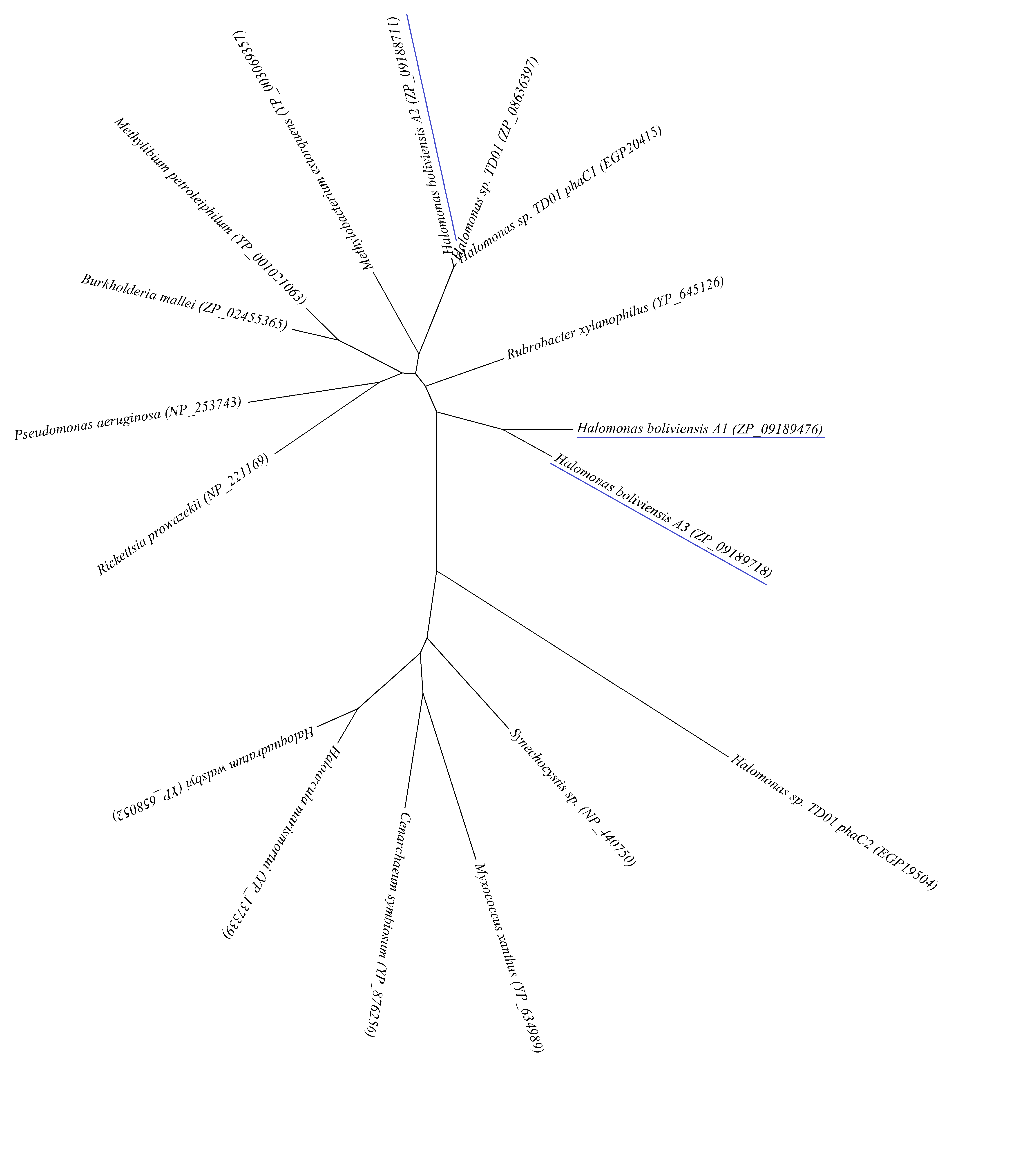

Supplement: Additional file 5 — Figure S1. [file 2046-9063-8-9-S5.TIFF]
